# The Relationship between Sleep Time and Mental Health Problems According to the Strengths and Difficulties Questionnaire in Children after an Earthquake Disaster: The Fukushima Health Management Survey

**DOI:** 10.3390/ijerph15040633

**Published:** 2018-03-30

**Authors:** Shuntaro Itagaki, Tetsuya Ohira, Masato Nagai, Seiji Yasumura, Masaharu Maeda, Yuriko Suzuki, Hirobumi Mashiko, Tetsuya Shiga, Itaru Miura, Hirooki Yabe

**Affiliations:** 1 Radiation Medical Science Center for the Fukushima Health Management Survey, Fukushima Medical University, Hikarigaoka-1, Fukushima 960-1295, Japan; teoohira@fmu.ac.jp (T.O.); mnagai@fmu.ac.jp (M.N.); yasumura@fmu.ac.jp (S.Y.); masagen@fmu.ac.jp (M.M.); yrsuzuki@ncnp.go.jp (Y.S.); mashiko@fmu.ac.jp (H.M.); tetsuya3@fmu.ac.jp (T.S.); itaru@fmu.ac.jp (I.M.); hyabe@fmu.ac.jp (H.Y.); 2 Department of Neuropsychiatry, School of Medicine, Fukushima Medical University, Hikarigaoka-1, Fukushima 960-1295, Japan; 3 Department of Epidemiology, School of Medicine, Fukushima Medical University, Hikarigaoka-1, Fukushima 960-1295, Japan; 4 Department of Public Health, School of Medicine, Fukushima Medical University, Hikarigaoka-1, Fukushima 960-1295, Japan; 5 Department of Disaster Psychiatry, School of Medicine, Fukushima Medical University, Hikarigaoka-1, Fukushima 960-1295, Japan; 6 Department of Adult Mental Health, National Institute of Mental Health, National Center of Neurology and Psychiatry, Tokyo 187-0031, Japan

**Keywords:** children, earthquake, mental health, nuclear reactor accident, sleep

## Abstract

A cross-sectional study was performed on the adverse effects of sleep time on the mental health of children after the Great East Japan Earthquake and subsequent nuclear reactor accident in March 2011. The target participants were children aged 4–15 years living inside the government-designated evacuation zone as of 11 March 2011 (*n* = 29,585). The participants’ parents/guardians completed the Strengths and Difficulties Questionnaire (SDQ) and sleep time data were obtained from the 2011 Fukushima Health Management Survey. A total of 18,745 valid responses were returned. We excluded questionnaires with incomplete answers leaving 13,272 responses for the final analysis. First, we divided the children into three age groups for analysis. Second, we divided each age group into four or five groups based on sleep time per day. We used SDQ scores ≥16 to indicate a high risk of mental health problems. In the 4–6-year-old group, those with a sleep time of <9 h had a higher risk. In the 7–12-year-old group, those with ≥10 h of sleep time had a higher risk. In the 13–15-year-old group, those with ≥9 h of sleep time had a higher risk. Shorter sleep time was associated with a higher risk of mental health in 4–6-year-olds. On the other hand, oversleeping was associated with a high risk of mental health in 7–15-year-olds.

## 1. Introduction

The effects of the Great East Japan Earthquake and subsequent nuclear accident at Tokyo Electric Power Company’s Fukushima Daiichi Nuclear Power Plant which occurred in March 2011, are expected to be long term, contributing to ongoing anxiety among the residents of Fukushima Prefecture. A 20 km-radius exclusion zone around the reactor and areas where the cumulative annual dose of radiation threatens to reach 20 mSv have been established by the government as planned evacuation zones; the residents of these areas have been forced to live either elsewhere in Fukushima or move outside the prefecture as evacuees. Fukushima Prefecture has been threatened by low-dose radioactivity since this nuclear accident occurred. Therefore, the citizens of this area have increased anxiety and stress. A major long-term health effect of the previous Chernobyl nuclear disaster has been the disruption of mental and physical processes [1]. In other words, anxieties over radiation, unexplained physical symptoms, and subjective health concerns have all been identified among residents in the exposed areas [2].

Soon after the disaster in Japan, Fukushima Prefecture launched the Fukushima Health Management Survey to investigate long-term low-dose radiation exposure caused by the accident. Fukushima Medical University took the lead in planning and implementing this survey, which had the primary purposes of monitoring the long-term health of residents, promoting their future wellbeing, and confirming whether long-term low-dose radiation exposure has health effects in humans [3].

In one section of the Fukushima Health Management Survey, the Mental Health and Lifestyle Survey, we have put health assistance into practice and developed insight into the lifestyle habits and health conditions of residents living in long-term shelters by studying and gaining an understanding of the various mental health factors affecting residents of the evacuated area [3]. This is important not only for the accumulation of scientific knowledge, but also for the provision of proper care.

There were several reports that sleep time had various effects on mental health. Pilcher and Huffcutt conducted a meta-analysis and reported that sleep deprivation strongly impairs human functioning and mood is more affected by sleep deprivation than either cognitive or motor performance [4]. Watson et al. reported that genetic contributions to depressive symptoms increase at both short and long sleep durations [5]. They reported that normal amount of 7 to 9 h sleep had a 27% heritability of depressive symptoms. But people who slept 5 h/night had 53% heritability and 10 h/night had 49% heritability [5]. Correlations between sleep disturbances and emotional/behavioral problems have been found to be statistically significant in a study that finally 912 people were recruited participants from the primary school in China [6]. Williams et al. reported that sleep problems in early childhood can help identify children at risk of poor school adjustment [7]. Usami et al. examined relationships between sleep duration and traumatic symptoms in children after the Great East Japan Earthquake and reported that those with evacuation experiences and/or whose homes had been damaged slept for a significantly shorter time after the tsunami experience in the survey 8 months after the earthquake [8].

The Strengths and Difficulties Questionnaire (SDQ) reveals both strengths and behavioral problems in children and can easily be completed by parents or teachers. The SDQ is an appropriate tool for screening the behavior and psychopathology of 3–16-year-old children [9]. Authorized translations of the SDQ are available free of charge and due to its ease of use, it has now been translated into more than 75 languages and validated in clinical and community samples [10,11].

No investigation has been performed on the relationship between sleep time and scores on SDQ after evacuation due to a nuclear plant accident. Therefore, we decided to examine the risk of mental health problems as indicated by SDQ in children after the Great East Japan earthquake disaster using data from the 2011 Fukushima Health Management Survey. 

The aim of this study was to examine the relationship between sleep time and risk of mental health problems after an earthquake disaster in children 4–15 years of age. We focused on the relationship between disaster and sleep time because disturbance of the sleeping habits of children has been associated with the risk of developing lifestyle diseases in the future, and examining the degree of risk is important not only to accumulate scientific knowledge, but also to provide appropriate care. This is the first large-scale report to examine the impact of sleep time in children in a nuclear accident environment.

## 2. Materials and Methods

### 2.1. Study Setting

This study is part of a longitudinal study to monitor the mental health status of evacuees of the Fukushima Nuclear Power Plant accident. Details are described elsewhere [12]. The investigation period is scheduled for 30 years after the disaster. In this paper, cross-sectional data collected in 2011 were analyzed [3]. In this survey, data collected from 31 October 2011 to 20 January 2012 were used. Investigators did not contact non-responders by telephone or any other method to request a response to the survey. All subjects gave their informed consent for inclusion before they participated in the study. The study was conducted in accordance with the Declaration of Helsinki, the protocol was approved by the Ethics Committee of the School of Medicine of Fukushima Medical University, Japan (approved code: 1316).

### 2.2. Participants

The overall cohort was the entire population of the evacuation zone designated by the government of Fukushima Prefecture (approximately 210,000 residents). Following the disaster, the government designated the 20-km radius around the Fukushima Daiichi Nuclear Power Plant a “restricted area” with compulsory evacuation. The government subsequently designated a 20- to 30-km area around the plant as an “evacuation-prepared area in case of emergency,” and areas near the 30-km radius where high-level radiation exposure was expected (>20 mSv/year) as “deliberate evacuation areas.” As a result, governmental direction forced all residents of Hirono Town, Naraha Town, Tomioka Town, Kawauchi Village, Okuma Town, Futaba Town, Namie Town, Katurao Town, and Iitate Village, to evacuate their homes; this was also the case for some areas of Tamura City, Minamisoma City, and Kawamata Town. The target participants were children aged 0–15 years as of 11 March 2011 (*n* = 29,585). We sent a questionnaire containing the SDQ to parents/guardians of children in the following three age groups: 0–6 years old, 6–12 years old, and 12–15 years old. A total of 18,745 valid responses were returned (response rate 63.4%) [3]. The SDQ was only administered to parents/guardians of children aged 4 to 15 years old; therefore, we used the data for children of this age group in our analysis (*n* = 17,868) [11]. We excluded questionnaires with incomplete answers from this study. Finally, responses from 13,272 people were included in the final analysis.

Participants were assigned a number. Sex, date of birth, address on 11 March 2011, and present address were collected from the survey. In children aged 4–15, we used the following items: experiencing the tsunami and nuclear reactor accident (i.e., heard the explosion) caused by the earthquake disaster, currently undergoing treatment for any illness, never been hospitalized due to illness, regular exercise apart from physical education class, and SDQ score. On the question sheet of the 12–15-year-old group, some answers including SDQ were self-report.

### 2.3. Assessments

The SDQ was completed and returned by the children’s parent/guardian. The SDQ is a brief, 25-item behavioral screening questionnaire that assesses the psychopathology and positive strengths of 3–16-year-old children. Each item is scored on a 3-point scale (0 = not true, 1 = somewhat true, 2 = definitely true). We used total SDQ scores as a measure of the strength and behavioral problems of children. There are several versions of the SDQ to meet the needs of researchers, clinicians and educators to measure behavioral and emotional problems [13]. The present study used the Japanese translation version. SDQ scores ≥16 were defined as high in accordance with a previous study that used 16 points as the cut-off for requiring mental health support [14]. The Japanese version of the SDQ has shown adequate internal consistency (α = 0.81) [11,14] and convergent validity [11].

### 2.4. Data Analysis

We categorized the age group into 3 groups for analysis. This categorization was adapted to Japanese age of school age. In other words, the preschool age was 4–6 years old, the elementary school students were 7–12 years old, and the junior high school was 13–15 years old in Japan. All analyses were conducted using SAS software (Version 9.3, Cary, NC, USA). With SDQ scores ≥16 as an outcome, nightly sleep time was segmented operationally and a chi-squared test (categorical variables), or analysis of variance (ANOVA; continuous variables) was performed. Secondly, we performed a Poisson regression analysis to determine the relationship between sleep time and mental health. We defined children with SDQ scores ≥16 as having the possibility of a mental health problem. A multivariate-adjusted Poisson regression analysis was used to calculate the risk ratio (RR) and 95% confidence intervals (95% CI) of mental health problems and sleep time after controlling simultaneously for potential confounders. Multivariate RRs were adjusted for age and sex plus current state of health (very good or good, normal, or poor or very poor), adequate sleep (adequate, slightly inadequate, or inadequate), evacuation (inside Fukushima or to other prefectures), tsunami experience (yes or no), nuclear reactor accident experience (yes or no), respondent of questions 1–6 (participant or proxy), and respondent of other questions (mother, father, or other).

## 3. Results

Table 1 shows the baseline characteristics of participants aged 4–6 years by sleep time. In this age group, sleep time was longer than in the higher age groups. Children in this age group with sleep time <9 h showed the highest mean SDQ scores. The majority of children aged 4–6 years had long sleep times with no napping. Participants that had evacuated to other prefectures slept for a long time. Tsunami experience and nuclear accident experience were not related to sleep time in the 4–6-year-old age group. Table 2 shows the baseline characteristics of participants aged 7–12 years by sleep time. Sleep time was shorter for females than males in this age group, and sleep time was shorter than the highest age group. Children in this age group with sleep time ≥10 h showed the highest mean SDQ scores. Similar to the 4–6-year-old age group, participants aged 7–12 years that had evacuated to other prefectures slept for a long time. Table 3 shows the baseline characteristics of participants aged 13–15 years by sleep time. As in the 7–12-year-old age group, sleep time was shorter for females than males. Mean SDQ score was high for those with both <6 h and ≥9 h sleep time in this age group. Sleep time was longer, and adequate sleep time was higher than in the younger age groups. Conversely, the sleep time was shorter, the inadequate sleep was higher than in the younger age groups.

RRs between sleep time and high risk of mental health problems as indicated by SDQ score ≥16 points by age group are shown in Table 4, Table 5 and Table 6. Table 4 shows the relationship between sleep time and mental health in the 4–6-year-old age group. In this age group, we used a sleep time of 9 h as a reference. Children aged 4–6 years old with sleep time <9 h had a high risk of mental health problems (multivariate RR = 1.22 (95% CI: 0.98−1.53)); however, according to *P* for trend, this relationship was not linear. Table 5 shows the relationship between sleep time and mental health problems in the 7−12-year-old age group. In this age group, we used a sleep time of 8 h sleep as a reference. Children aged 7−12 years old with longer sleep times had a high risk of mental health problems (9 h: multivariate RR = 1.12 (95% CI: 1.02–1.24); ≥10 h: multivariate RR = 1.30 (95% CI: 1.12–1.51)). Table 6 shows the relationship between sleep time and mental health in the 13–15-year-old age group. In this age group, we used a sleep time of 7 h as a reference. Children aged 13–15 years old with both short and long sleep times had a high risk of mental health problems as evaluated by SDQ (<6 h: multivariate RR = 1.13 (95% CI: 0.82–1.56); 8 h; multivariate RR = 1.19 (95% CI: 0.93–1.51); ≥9 h: multivariate RR = 2.04 (95% CI: 1.49–2.77)).

## 4. Discussion

Our finding of sleep times becoming shorter with age agrees with the results of previous studies [15,16]. Participants in the 4–6-year-old and 7–12-year-old age groups that had evacuated to other prefectures slept for a longer time than those who evacuated inside Fukushima Prefecture. On the other hand, tsunami experience and nuclear accident experience were not related to sleep time. This fact presumes that the evacuation life may be more stressful than experiencing the tsunami and nuclear accident. In any age groups, regardless of sleeping time, it was majority that the current state of health state was very good, good, or normal. First, we speculated that because of “current state of health” means “physical health”, it did not change regardless of sleep state. Second, it may be related to the cultural and social factors. Unlike surveys after disasters all over the world, Hori et al. reported that in 2011 after 3.11 new outpatients of the depressive episode or other mood disorders in Fukushima Prefecture had decreased visits and estimated the cultural background [17]. They speculated that altruistic feelings of unity and nationalistic eagerness for recovery prevailed in Japan after disaster, and many residents in disaster-stricken areas may have had a heightened sense of purpose to overcome the threat of radiation exposure and to rebuild their hometowns [17].

Biggs et al. reported that inconsistent sleep schedules significantly increased the risk for behavioral difficulties according to SDQ scores in school-aged children [18]. Wu et al. also reported that preschool children with short nighttime sleep duration were significantly more likely to have behavioral problems than those with longer sleep duration [19]. Matamura et al. reported that a late bedtime and short sleep duration could predict subsequent development of depression and anxiety, including suicidal or self-injury risk [20]. Similar to previous studies, our study also showed that children aged 4–6 years a with short sleep time had a high risk of mental health problems [18,19,20].

There was a report that long sleep affects metabolically unhealthy normal weight as much as short sleep [21]. Cappuccio et al. reported that both long sleep and short sleep were associated with a greater risk of death by meta-analysis [22]. There are reports that overnight sleep time on the weekend affects behavior disorder, academic performance, mood, anxiety, and drug dependence [23,24,25,26]. However, to the best of our knowledge, there have been no reports that long sleep times over the long-term adversely affect mental health as indicated by SDQ. We speculate that the survey was carried out 7 to 11 months after the disaster, so that there were many environmental factors. For example, long-sleeping people of 4–6 and 7–12 years old were more frequently evacuated to other prefectures. The present study showed that 13–15-year-olds with both short sleep time and oversleeping had a high risk of mental health problems. Extreme sleep time as short or long may adversely affect mental health.

One limitation of this research is that because family members completed the questionnaire for participants aged 4–12 years old, responses may indicate bedtime and not actual sleep time. We investigated bedtime and wake-up time, but it may be that they were listening to activity reduction rather than sleeping time. Part of participants aged 13–15 years old completed the questions about sleep time themselves, their responses are thought to represent actual sleep time. Another limitation is that we did not distinguish between sleeping behavior on weekdays and weekends and we did not investigate the quality of sleep. This is also a factor that may not be able to confirm the actual sleep behavior.

## 5. Conclusions

Previous studies have reported that suboptimal sleep patterns were associated with an array of mental disorders and other health-related outcomes among adolescents. This is the first large-scale report to examine the impact of sleep time among children in a nuclear accident environment. Determining the relationship between the health of disaster victims and sleep time is valuable because disturbance of sleeping habits in children has been associated with a high risk of mental health problems in the future, and examining the degree of risk is important not only for the accumulation of scientific knowledge, but also as for sleep guidance and providing appropriate care to disaster victims. In our survey after the disaster, it was shown that young people’s short sleep and long sleep worsen the health indicated by SDQ. This result suggested the importance of an appropriate amount of sleep behavior in support after a large-scale disaster.

## Figures and Tables

**Table 1:** Baseline characteristics of participants aged 4–6 years by sleep time.

	Sleep Time (h/day)				*p* Value ^a^
	<9	9	10	≥11	
Participants (*n*)	191	1368	1530	294	
Female (%)	51.3	48.6	49.5	51.7	0.742
Mean age (years) (SD)	5.2 (0.8)	5.1 (0.8)	4.9 (0.8)	4.7 (0.8)	<0.0001
Mean SDQ (SD)	13.1 (6.4)	11.7 (5.6)	11.7 (5.7)	12.0 (5.7)	0.012
Current state of health (%)					
Very good or good	47.1	55.1	57.8	51.0	0.054
Normal	51.3	43.4	40.3	47.3	
Poor or very poor	1.6	1.6	1.9	1.7	
Napping time (%)					
None	44.4	56.7	71.8	82.6	<0.0001
<1 hour/day	2.7	1.8	1.8	1.0	
1−2 hours/day	25.9	22.5	16.5	11.3	
≥2 hours/day	27.0	18.9	9.9	5.1	
Tsunami experience (%)	8.4	9.7	9.4	10.2	0.914
Nuclear reactor accident experience (%)	39.3	33.9	34.3	39.1	0.188
Evacuation to other prefectures (%)	15.7	27.3	39.3	47.3	<0.0001
Respondent (%)					
Mother	88.5	91.6	92.8	91.5	0.206
Father	10.5	7.5	5.8	7.5	
Other	1.1	1.0	1.4	1.0	

SD, standard deviation; ^a^ *p* values were calculated by a chi-squared test (categorical variables), or ANOVA (continuous variables).

**Table 2:** Baseline characteristics of participants aged 7–12 years by sleep time.

	Sleep Time (h/day)				*p* Value ^a^
	<8	8	9	≥10	
Participants (*n*)	777	2758	3322	593	
Female (%)	58.6	49.5	46.5	47.7	<0.0001
Mean age (years) (SD)	12.2 (1.1)	10.9 (1.6)	9.7 (1.5)	9.2 (1.4)	<0.0001
Mean SDQ (SD)	10.4 (6.3)	10.6 (6.1)	11.1 (6.2)	12.2 (6.8)	<0.0001
Current state of health (%)					
Very good or good	49.2	53.4	55.0	52.7	2 × 10^−4^
Normal	47.1	43.0	43.1	43.4	
Poor or very poor	3.6	3.6	1.9	3.9	
Tsunami experience (%)	11.6	12.4	11.4	11.3	0.618
Nuclear reactor accident experience (%)	42.7	41.4	37.7	40.0	0.007
Evacuation to other prefectures (%)	21.9	22.5	29.7	47.9	<0.0001
Respondent (%)					
Mother	86.6	89.1	88.1	89.4	0.378
Father	11.3	9.2	9.6	8.9	
Other	2.1	1.7	2.3	1.7	

SD, standard deviation; ^a^ *p* values were calculated by a chi-squared test (categorical variables), or ANOVA (continuous variables).

**Table 3:** Baseline characteristics of participants aged 13–15 years by sleep time.

	Sleep Time (h/day)					*p* Value ^a^
	<6	6	7	8	≥9	
Participants (*n*)	160	675	932	557	115	
Female (%)	63.8	55.4	50.0	42.9	35.7	<0.0001
Mean age (years) (SD)	14.6 (0.6)	14.5 (0.6)	14.4 (0.6)	14.3 (0.6)	14.3 (0.6)	<0.0001
Mean SDQ (SD)	11.9 (6.6)	9.9 (6.2)	9.4 (5.6)	9.9 (6.3)	12.6 (7.0)	<0.0001
Current state of health (%)						
Very good or good	35.4	46.3	53.5	54.8	45.6	<0.0001
Normal	53.2	48.1	42.8	40.3	50.0	
Poor or very poor	11.4	5.7	3.8	4.9	4.4	
Adequate sleep (%)						
Adequate	10.6	22.9	44.1	71.0	70.4	<0.0001
Slightly inadequate	45.6	59.1	46.2	24.1	22.6	
Inadequate	43.8	18.0	9.8	4.9	7.0	
Tsunami experience (%)	14.4	14.7	11.1	14.8	12.5	0.212
Nuclear reactor accident experience (%)	51.0	42.6	42.7	42.8	37.5	0.254
Evacuation to other prefectures (%)	18.8	20.3	20.7	21.9	20.9	0.921
Respondent of Q1−6 (%)						
Participant	78.7	76.0	74.7	73.1	71.6	0.526
Proxy	21.3	24.0	25.3	26.9	28.4	
Respondent of other questions (%)						
Mother	87.4	83.9	84.6	84.1	79.0	0.294
Father	10.7	13.7	13.4	13.0	14.9	
Other	1.9	2.4	2.0	2.9	6.1	

SD, standard deviation; ^a^ *p* values were calculated by a chi-squared test (categorical variables), or ANOVA (continuous variables).

**Table 4:** The relationship between sleep time and mental health in participants aged 4–6 years.

	Sleep Time (h/day)				*p* for Trend ^a^
	<9	9	10	≥11	
Participants (*n*)	191	1368	1530	294	
SDQ ≥16 (*n*)	59	328	368	72	
Crude RR	1.29 (1.02−1.63)	1.00 (reference)	1.00 (0.88−1.14)	1.02 (0.82−1.28)	0.156
Age-sex-adjusted RR ^b^	1.30 (1.04−1.64)	1.00 (reference)	0.98 (0.86−1.12)	0.97 (0.78−1.21)	0.053
Multivariate RR ^c^	1.22 (0.98−1.53)	1.00 (reference)	1.01 (0.89−1.15)	0.96 (0.77−1.21)	0.131

RR, risk ratio; ^a^ *p* for trend values were calculated with sleep time as a continuous variable; ^b^ Adjusted for sex (male or female) and age (4 years old, 5 years old, or 6 years old); ^c^ Adjusted for age and sex plus current state of health (very good or good, normal, or poor or very poor), napping time (none, <1 h/day, 1−2 h/day, or ≥2 h/day), evacuation (inside Fukushima or to other prefectures), tsunami experience (yes or no), nuclear reactor accident experience (yes or no), and respondent (mother, father, or other).

**Table 5:** The relationship between sleep time and mental health in participants aged 7–12 years.

	Sleep Time (hours/day)				*p* for Trend ^a^
	<8	8	9	≥10	
Participants (*n*)	777	2758	3322	593	
SDQ ≥16 (*n*)	152	552	759	171	
Crude RR	0.98 (0.83−1.15)	1.00 (reference)	1.14 (1.04−1.26)	1.44 (1.24−1.67)	<0.0001
Age-sex-adjusted RR ^b^	1.02 (0.87−1.20)	1.00 (reference)	1.09 (0.98−1.20)	1.34 (1.16−1.57)	0.015
Multivariate RR ^c^	0.99 (0.85−1.16)	1.00 (reference)	1.12 (1.02−1.24)	1.30 (1.12−1.51)	0.002

RR, risk ratio; ^a^ *p* for trend values were calculated with sleep time as a continuous variable; ^b^ Adjusted for sex (male or female) and age (7 years old, 8 years old, 9 years old, 10 years old, 11 years old, and 12 years old); ^c^ Adjusted for age and sex plus current state of health (very good or good, normal, or poor or very poor), evacuation (inside Fukushima or to other prefectures), tsunami experience (yes or no), nuclear reactor accident experience (yes or no), and respondent (mother, father, or other).

**Table 6:** The relationship between sleep time and mental health in participants aged 13–15 years.

	Sleep Time (h/day)					*p* for Trend ^a^
	<6	6	7	8	≥9	
Participants (*n*)	160	675	932	557	115	
SDQ ≥16 (*n*)	39	109	136	95	36	
Crude RR	1.67 (1.22−2.29)	1.11 (0.88−1.40)	1.00 (reference)	1.17 (0.92−1.49)	2.15 (1.57−2.93)	0.580
Age-sex-adjusted RR ^b^	1.67 (1.22−2.30)	1.11 (0.88−1.40)	1.00 (reference)	1.17 (0.92−1.49)	2.14 (1.56−2.94)	0.580
Multivariate RR ^c^	1.13 (0.82−1.56)	0.97 (0.77−1.22)	1.00 (reference)	1.19 (0.93−1.51)	2.04 (1.49−2.77)	0.015

RR, risk ratio; ^a^ *p* for trend values were calculated with sleep time as a continuous variable; ^b^ Adjusted for sex (male or female) and age (13 years old, 14 years old, or 15 years old); ^c^ Adjusted for age and sex plus current state of health (very good or good, normal, or poor or very poor), adequate sleep (adequate, slightly inadequate, or inadequate), evacuation (inside Fukushima or to other prefectures), tsunami experience (yes or no), nuclear reactor accident experience (yes or no), respondent of Q1–6 (participant or proxy), respondent of other questions (mother, father, or other).
